# Supplementary material for: No general stability conditions for marine ice-sheet grounding lines in the presence of feedbacks
Source: Nat Commun. 2022 Apr 27;13:2265. doi: 10.1038/s41467-022-29892-3 (PMC9046188; doi:10.1038/s41467-022-29892-3)
Supplement: Supplementary file 1 — Supplementary Information [file 41467_2022_29892_MOESM1_ESM.pdf]

Supplementary Information

No general stability conditions for marine ice-sheet grounding lines in the presence of feedbacks

O. V. Sergienko

Program in Atmospheric and Oceanic Sciences, Princeton University, Princeton, USA

email: osergien@princeton.edu

Supplementary Methods 1 Numerical simulations

All numerical simulations are performed with the finite-element solver Comsol™[1]. The steady-state solutions are obtained by solving an optimization problem using a minimization procedure based on the Bound Optimization by Quadratic Approximation optimization algorithm[2]. In all simulations, the grid resolution is spatially variable: it is 200 m through 95% of the length of the domain, and 1 m in the 5% closest to the grounding line position x_g.

Parameters in expression (1) of the main text are the following: a_1=2.4 m yr^{-1}, a_2=0.8 m yr^{-1}, T_0=-15 °C and σ=6°C. We use T_{sl}=-4°C in Eqn. (2) of the main text. In all simulations the bed elevation is $B(x) = b_0 + b_a cos\frac{\pi x}{L}$, with b_0=-500 m, b_a=250 m and L=500 km; sliding law parameters are C=7.6·10^6 Pa m$^{-1/3}$s$^{1/3}$; $m = 1/n = 1/3$.

In time-variant simulations the time-dependent problem

$$2\left(A^{-1/n} h \left|u_x\right|^{1/n-1} u_x\right)_x - \tau_b - \rho g h S_x = 0 \quad \text{for} \quad x_d \le x \le x_g(t), \tag{1a}$$

$$h_t + (uh)_x = \dot{a} \quad \text{for} \quad x_d \le x \le x_g(t), \tag{1b}$$

$$S_x = 0, \quad u = 0 \quad \text{at} \quad x = x_d, \tag{1c}$$

$$2A^{-1/n} h \left|u_x\right|^{1/n-1} u_x = \frac{1}{2}\rho g' h^2 \quad \text{at} \quad x = x_g(t), \tag{1d}$$

$$h = -\frac{\rho_w}{\rho} B \quad \text{at} \quad x = x_g(t). \tag{1e}$$

is solved iteratively using a direct solver (*i.e.*, the momentum and mass-balance equations (1a) and (1b), along with the corresponding boundary conditions (1c)-(1e) are solved simultaneously) at every time step. The model is initialized with a prescribed grounding line position $x_g(t = 0) = \hat{x}_g - 1$ km, where $\hat{x}_g$ is the location of the steady-state grounding line. The initial conditions at $t = 0$ are obtained by solving (1) iteratively using steady-state solutions of the ice thickness $h(0 < x \leq x_g(t = 0))$ and ice velocity $u(0 < x \leq x_g(t = 0))$ as an initial guess for the iterative procedure. Iterations stop when the residual (the difference between the terms of the left-hand side and the right-hand side of the problem (1)) becomes smaller than the relative tolerance 10^{-6}. The distributions $h(x, t = 0)$ and $u(x, t = 0)$ are shown in fig. 5. Note that the ice thickness at the perturbed position of the grounding line (red line in the inset in fig. 5(a)) is larger than the ice thickness at the steady-state grounding line position (blue line) in accordance with the flotation condition (1e). The grounding lines are on the up-sloping part of the bed, and the perturbed, upstream position is on a slightly deeper part of the bed. These initial configurations are no longer in steady states.

In both cases, $\dot{a} = \dot{a}(T_S(S))$ and $\dot{a} = \dot{a}(x)$, the grounding lines begin to advance from the perturbed positions towards the steady-state positions (Fig. 3 of the main text). In the case of $\dot{a} = \dot{a}(T_S(S))$, $\dot{a}$ evolves in time because the ice-sheet surface evolves in time as the grounding

line advances. Fig. 6a shows snapshots of the evolving ice-sheet surface and Fig. 6b shows the corresponding spatial distributions of $\dot{a}(T_S(S))$ (note different time-intervals for the first three snapshots). The distribution of $\dot{a}(T_S(S))$ shown in Fig. 4 does not change with time and is the same for steady-state and time variant simulations. In the case of $\dot{a} = \dot{a}(x)$, the spatial distribution does not change with time and is the same as shown in Fig. 2b of the main text.

The eigenvalues in Fig. 4a of the main text are obtained by solving numerically the linear perturbation problem (8) with $\dot{a}_S$ computed from Eqns. (1)-(2) of the main text. The eigenvalues in Fig. 4b of the main text are obtained by solving (8) with $\dot{a}_S(\hat{s})$=0.

Supplementary Methods 2 Linear stability analysis

We consider the time-variant version of the approximate problem (5) described in the **Methods** of the main text

$$-C\frac{|q|^{m-1}q}{h^m} - \rho g h S_x = 0, \tag{2a}$$

$$h_t + (uh)_x = \dot{a}(s) \tag{2b}$$

$$(h+B)_x = 0, \quad u = 0 \quad x = x_d \tag{2c}$$

$$(\dot{a} - h_t)h^{m+2} + \frac{C}{\rho g}q^{m+1} + qh^{m+1}B_x = \left(\frac{A^{\frac{1}{n}}}{4}\rho g'\right)^n h^{n+m+3} \quad \text{at } x = x_g \tag{2d}$$

$$h = -\frac{\rho_w}{\rho}B \quad \text{at } x = x_g, \tag{2e}$$

where $\dot{a}(s) = \dot{a}(T_s(s))$ is defined by eqn. (1) and T_s by eqn. (2) and $S = B + h$. Considering small perturbations around the steady state,

$$h = \hat{h}(x) + \sigma\tilde{h}(x,t), \quad q = \hat{q}(x) + \sigma\tilde{q}(x,t), \quad S = \hat{S} + \sigma\tilde{S}(x,t)$$
$$\dot{a}(s) = \dot{a}(\hat{S}) + \sigma\dot{a}_S(\hat{S})\tilde{S}(x,t), \quad x_g = \hat{x}_g + \sigma\tilde{x}_g(t), \tag{3}$$

where we denote steady states by a symbol $\hat{}$. Assuming that the bed elevation B does not vary, $\tilde{s} = \tilde{h}$. Substituting these expressions to (2) leads to the perturbation problem to the lowest order in σ

$$m\frac{C}{\rho g}\hat{q}^{m-1}\tilde{q} + \hat{h}^{m+1}\tilde{h}_x + (m+1)\hat{h}^m\tilde{h}(\hat{h}+B)_x = 0 \tag{4a}$$

$$\tilde{h}_t + \tilde{q}_x = \dot{a}_s(\hat{s})\tilde{h} \tag{4b}$$

$$\tilde{q} = 0, \tilde{h}_x = 0, \quad x = 0 \tag{4c}$$

$$\begin{aligned}&(m+2)\hat{h}^{m+1}\tilde{h}\hat{q}_x + \tilde{q}_x\hat{h}^{m+2} + (m+1)\frac{C}{\rho g}\hat{q}^m\tilde{q} + \tilde{q}\hat{h}^{m+1}B_x + (m+1)\hat{h}^m\tilde{h}\hat{q}B_x\\ &+ \tilde{x}_g\left[\hat{h}^{m+2}\hat{q}_x + \frac{C}{\rho g}\hat{q}^{m+1} + \hat{q}\hat{h}^{m+1}B_x\right]_x\\ &= \left(\frac{A^{\frac{1}{n}}}{4}\rho g'\right)^n(m+n+3)\hat{h}^{n+m+2}\tilde{h} + \left(\frac{A^{\frac{1}{n}}}{4}\rho g'\right)^n\tilde{x}_g\left[\hat{h}^{n+m+3}\right]_x\end{aligned} \tag{4d}$$

$$\tilde{h} + \hat{h}_x\tilde{x}_g = -\frac{B_x}{1-\delta}\tilde{x}_g, \quad x = \hat{x}_g. \tag{4e}$$

From (4a) and (2a)

$$\tilde{q} = \frac{m+1}{m}\frac{\hat{q}}{\hat{h}}\tilde{h} - \frac{1}{m}\frac{\rho g}{C}\frac{\hat{h}^{m+1}}{\hat{q}^{m-1}}\tilde{h}_x. \tag{5}$$

Substituting this expression into (4b) leads to a parabolic partial differential equation (PDE)

for $\tilde{h}$

$$\tilde{h}_t + \left[\frac{m+1}{m}\frac{\hat{q}}{\hat{h}}\tilde{h} - \frac{1}{m}\frac{\rho g}{C}\frac{\hat{h}^{m+1}}{\hat{q}^{m-1}}\tilde{h}_x\right]_x = \dot{a}_S(\hat{S})\tilde{h}. \tag{6}$$

Using the method of separation of variables one arrives at the form

$$\tilde{h}(x,t) = \tilde{h}(x)e^{\Lambda t}. \tag{7}$$

Substituting $\tilde{h}$ in this form into (6) and writing (4b) as $\tilde{q}_x = \dot{a}_S(\hat{S})\tilde{h} - \Lambda\tilde{h}$ gives

$$\left[\frac{m+1}{m}\frac{\hat{q}}{\hat{h}}\tilde{h} - \frac{1}{m}\frac{\rho g}{C}\frac{\hat{h}^{m+1}}{\hat{q}^{m-1}}\tilde{h}_x\right]_x - \dot{a}_S(\hat{S})\tilde{h} = -\Lambda\tilde{h} \tag{8a}$$

$$\tilde{h}_x = 0, \quad x = 0 \tag{8b}$$

$$-\Lambda\tilde{h}A_1 + \tilde{q}A_2 = \tilde{h}\left[A_3 - A_4\right]. \tag{8c}$$

where

$$A_1 = \hat{h}^{m+2} \tag{9a}$$

$$A_2 = (m+1)\frac{C}{\rho g}\hat{q}^m + \hat{h}^{m+1}B_x \tag{9b}$$

$$A_3 = \left(\frac{A^{\frac{1}{n}}}{4}\rho g'\right)^n (m+n+3)\hat{h}^{n+m+2} - (m+2)\hat{h}^{m+1}q_x - (m+1)\hat{h}^m\hat{q}B_x - \dot{a}_S(\hat{S})\hat{h}^{m+2} \tag{9c}$$

$$A_4 = \frac{\tilde{1}}{h_x + \frac{B_x}{1-\delta}}\left\{\left[\left(\frac{A^{\frac{1}{n}}}{4}\rho g'\right)^n \hat{h}^{n+m+3}\right]_x - \left[\hat{h}^{m+2}q_x + \frac{C}{\rho g}\hat{q}^{m+1} + \hat{q}\hat{h}^{m+1}B_x\right]_x\right\} \tag{9d}$$

which is a second-order eigenvalue problem with Λ being the eigenvalue. It is possible to put (8) into a Sturm-Liouville form

$$\left[\mu(x)P(x)\tilde{h}_x\right]_x - \mu(x)R(x)\tilde{h} = \Lambda\tilde{h}, \tag{10}$$

through using the integrating factor

$$\mu(x) = \frac{\rho g}{C} \frac{e^{\int^x \frac{F(x)}{P(x)} dx}}{P(x)} \tag{11}$$

where

$$P(x) = \frac{\hat{h}^{m+1}}{m\hat{q}^{m-1}} \tag{12a}$$

$$R(x) = \frac{m+1}{m} \left(\frac{\hat{q}}{\hat{h}} \right)_x - \dot{a}_S(\hat{S}) \tag{12b}$$

$$F(x) = \frac{1}{m} \left[\frac{\rho g}{C} \left(\frac{\hat{h}^{m+1}}{\hat{q}^{m-1}} \right)_x - (m+1)\frac{\hat{q}}{\hat{h}} \right] \tag{12c}$$

From (4b)

$$\tilde{q}(x_g) = -\Lambda \int_0^{x_g} \tilde{h} dx + \int_0^{x_g} \tilde{h} \dot{a}_S(\hat{S}(x)) dx \tag{13}$$

Substituting this expression to (8c), rearranging terms and dividing both sides by $\tilde{h}$ gives

$$\Lambda = -\left[\hat{h}^{m+2} + \left(m\frac{C}{\rho g}\hat{q}^m - \hat{h}^{m+1} h_x \right) \frac{\int_0^{x_g} \tilde{h} dx}{\tilde{h}} \right]^{-1} \left[h_x + \frac{B_x}{1-\delta} \right]^{-1}$$
$$\times \left[\frac{B_x}{1-\delta} \left\{ \left(\frac{A^{\frac{1}{n}}}{4} \rho g' \right)^n (m+n+3)\hat{h}^{n+m+2} - \dot{a}(m+2)\hat{h}^{m+1} - (m+1)\hat{q}\hat{h}^m B_x \right\} + \right.$$
$$\left\{ - \frac{\int_0^{x_g} \tilde{h} \dot{a}_S(\hat{S}(x)) dx}{\tilde{h}} \left(h_x + \frac{B_x}{1-\delta} \right) \left(m\frac{C}{\rho g}\hat{q}^m - \hat{h}^{m+1} h_x \right) + \hat{h}^{m+2} \dot{a}_S(\hat{S}) h_x \right.$$
$$\left. \left. + \dot{a} \left[(m+1)\frac{C}{\rho g}\hat{q}^m + \hat{h}^{m+1} B_x \right] + \hat{q}\hat{h}^{m+1} B_{xx} \right\} \right]. \tag{14}$$

If $\dot{a}(x)$ is a function of x only as considered in ref.[3]

$$\Lambda = -\left[\hat{h}^{m+2} + \left(m\frac{C}{\rho g}\hat{q}^m - \hat{h}^{m+1}h_x\right)\frac{\int_0^{x_g}\tilde{h}dx}{\tilde{h}}\right]^{-1}\left[h_x + \frac{B_x}{1-\delta}\right]^{-1}$$
$$\times\left[\frac{B_x}{1-\delta}\left\{\left(\frac{A^{\frac{1}{n}}}{4}\rho g'\right)^n (m+n+3)\hat{h}^{n+m+2} - \dot{a}(m+2)\hat{h}^{m+1} - (m+1)\hat{q}\hat{h}^m b_X\right\} +\right.$$
$$\left.+\left\{\hat{h}^{m+2}\dot{a}_x + \dot{a}\left[(m+1)\hat{q}^m + \hat{h}^{m+1}B_x\right] + \hat{q}\hat{h}^{m+1}B_{xx}\right\}\right]. \qquad (15)$$

The term $\frac{\int_0^{x_g}\tilde{h}dx}{\tilde{h}}$ >0, according to theorem 1 of ref.[4]. This allows to infer a sign of the first term in square brackets in (14). However, it is not possible to determine the term in the second curly brackets on the right-hand side that includes $\frac{\int_0^{x_g}\tilde{h}\dot{a}_S(\hat{S}(x))dx}{\tilde{h}}$ without solving the eigenvalue problem to determine $\tilde{h}(x)$. Consequently, it is not possible to make an inference of the sign of Λ based on the properties of the steady-state solutions (expressions with $\hat{}$) at the steady-state grounding line position $\hat{x}_g$. As apparent from these considerations, this result – inability to determine a general stability condition based on properties of a steady state – is independent of a specific form or other details of the functional dependence of net accumulation/ablation rate on the surface temperature and hence surface elevation.

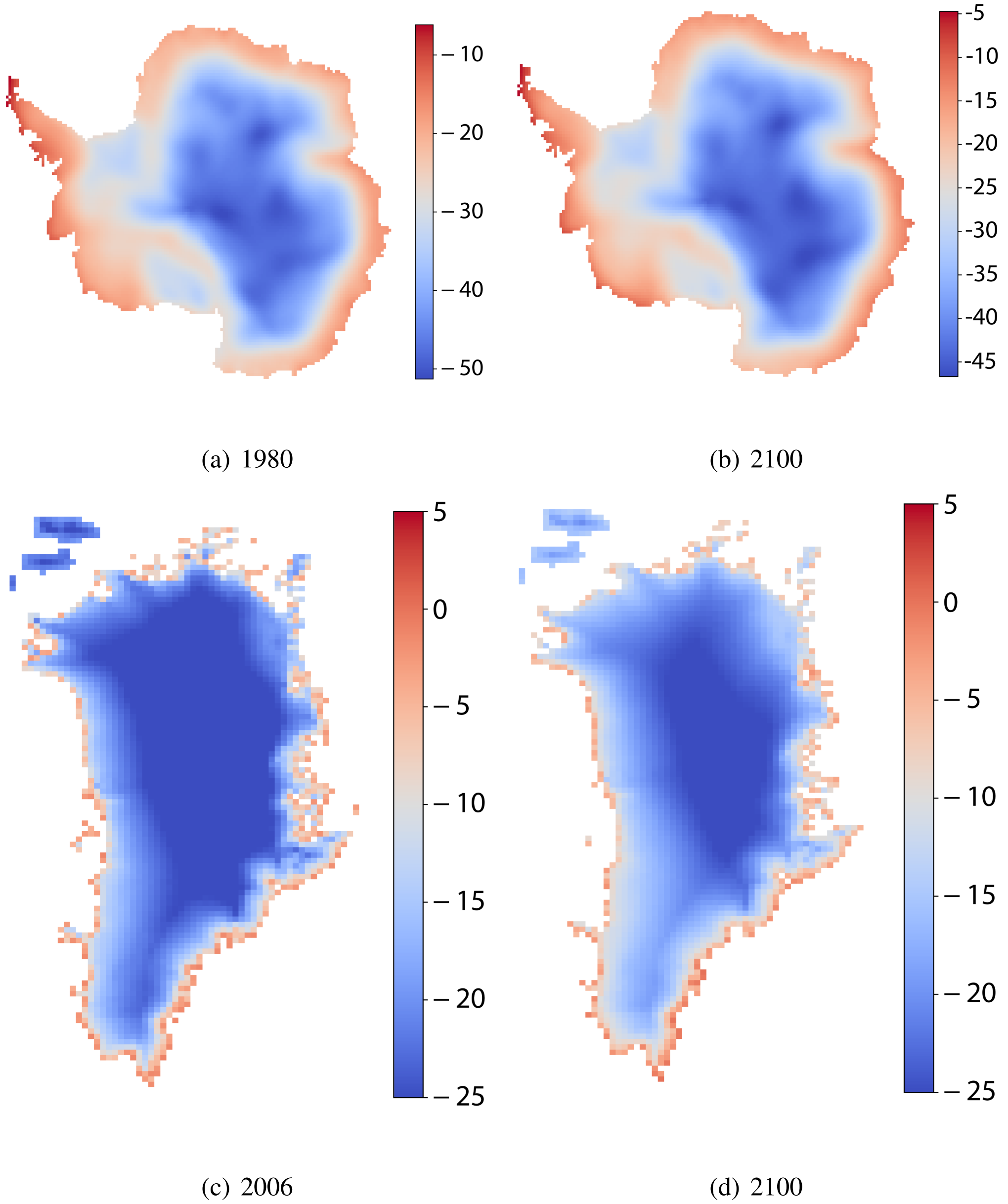

(a) 1980 (b) 2100

(c) 2006 (d) 2100

Figure 1: Annual mean surface temperature T (°C) simulated by MAR for the RCP8.5 scenario. (a)-(b) Antarctica; (c)-(d) Greenland.

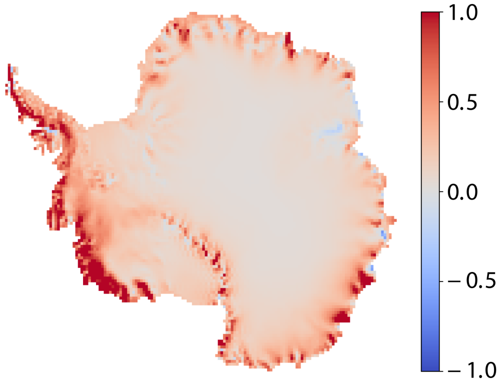

(a) 1980

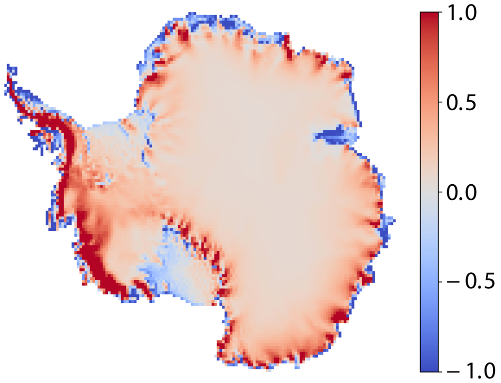

(b) 2100

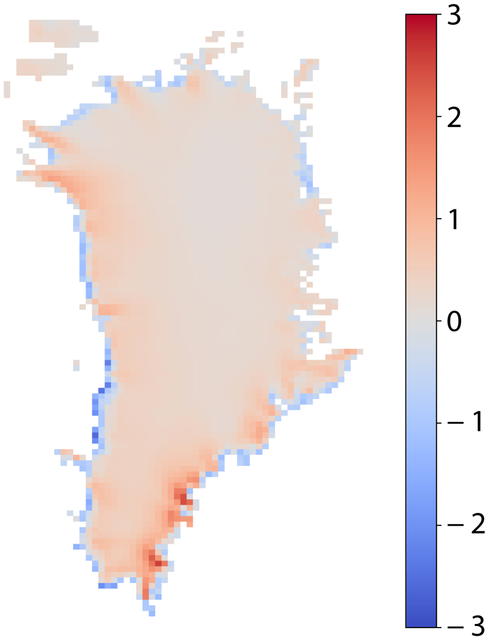

(c) 2006

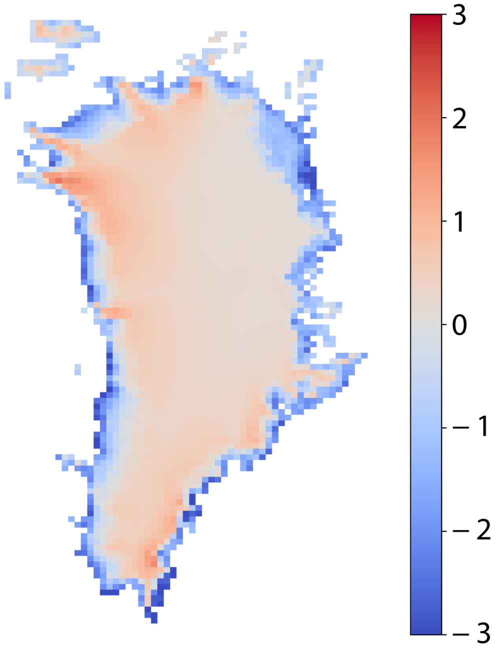

(d) 2100

Figure 2: Annual accumulation/ablation rate $\dot{a}$ (m yr^{-1}) simulated by MAR for RCP8.5 scenario. (a)-(b) Antarctica [5]; (c)-(d) Greenland [6].

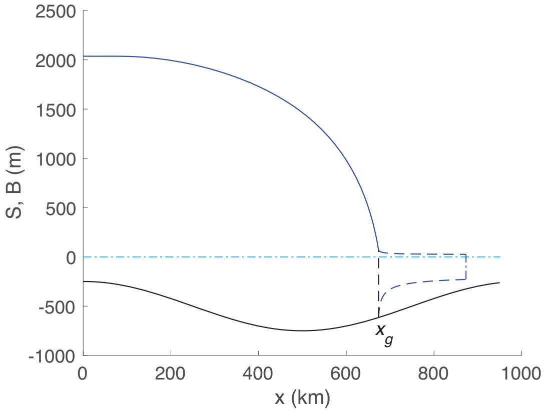

(a) S, B (m)

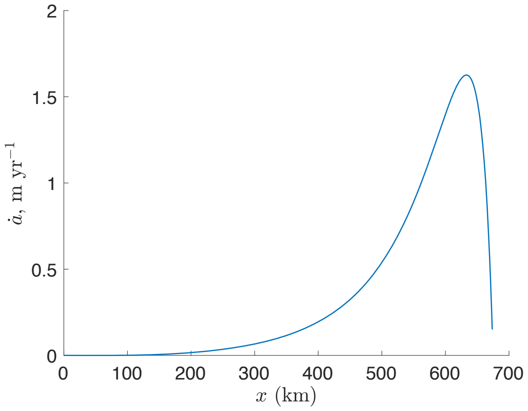

(b) $\dot{a}(x)$, (m yr^{-1})

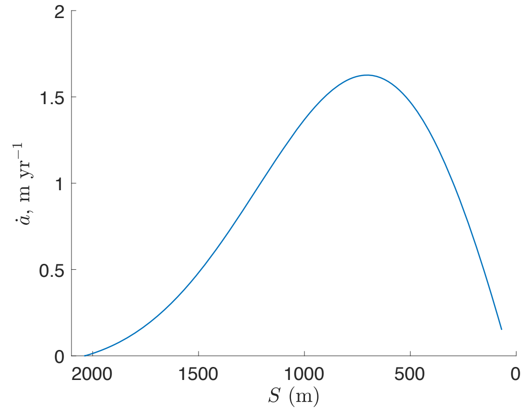

(c) $\dot{a}(S)$, (m yr^{-1})

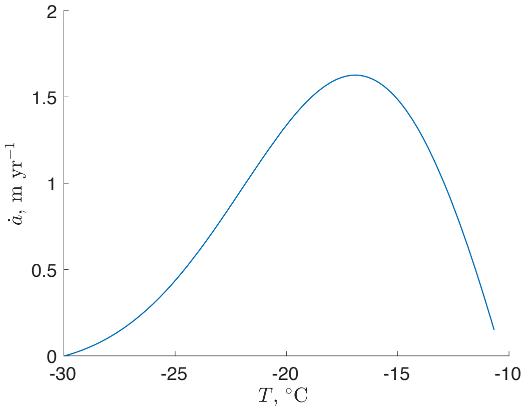

(d) $\dot{a}(S)$, (m yr^{-1})

Figure 3: Steady-state configuration for T_{sl}=-10 °C. (a) surface S and bed (B) elevation (m); accumulation/ablation rate $\dot{a}$ (m yr^{-1}) *vs* (b) distance from the divide x (km); (c) ice-sheet surface elevation S (m) (note the reverse direction of the horizontal axis); (d) ice-sheet surface temperature T_S (°C).

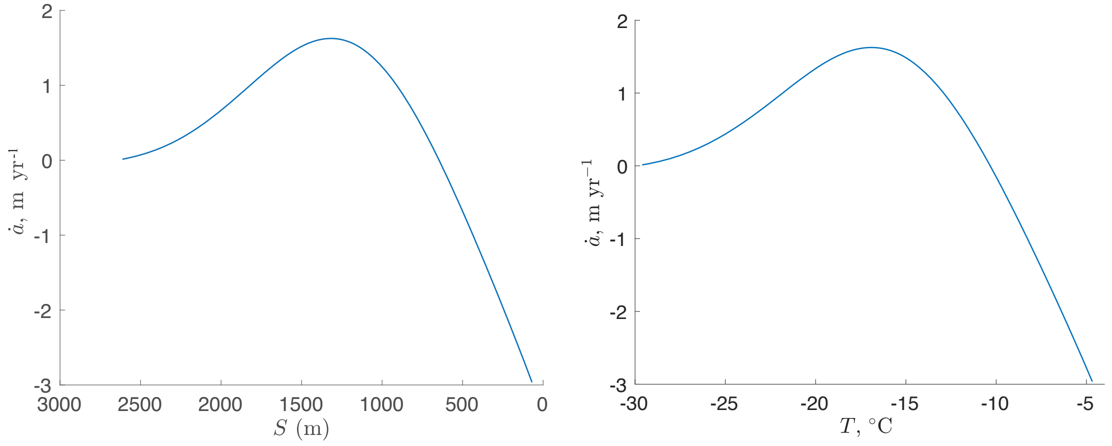

(a) $\dot{a}(S)$ (b) $\dot{a}(T_S)$

Figure 4: Accumulation/ablation rate $\dot{a}$ (m yr^{-1}) *vs* (a) ice-sheet surface elevation S (m) note the reverse direction of the horizontal axis); (b) ice-sheet surface temperature T_S (°C) for the steady-state ice-sheet configuration shown in Fig. 1 of the main text, T_{sl}=-4 °C.

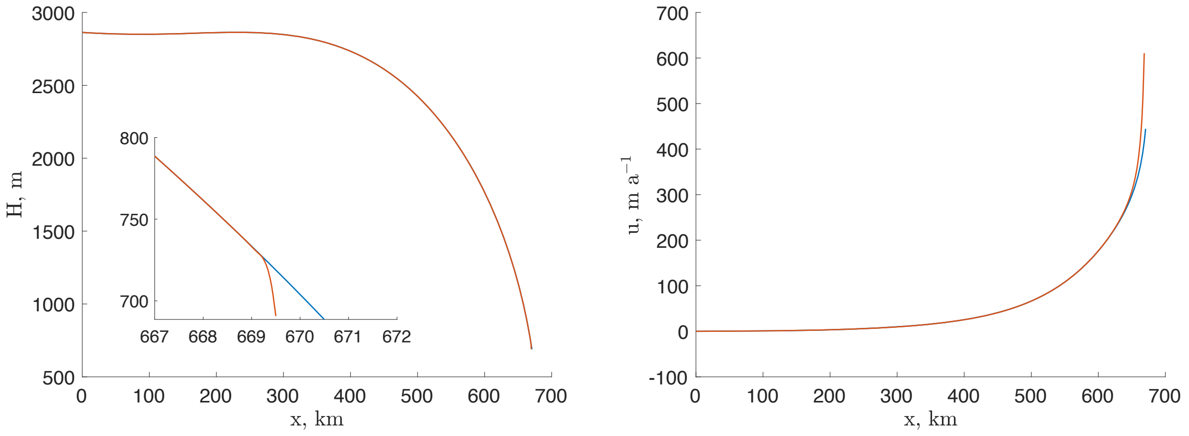

(a) $h(x, t = 0)$, m (b) $u(x, t = 0)$, m yr^{-1}

Figure 5: Initial conditions for the time-dependent simulations (red lines). (a) ice thickness $h(x, t = 0)$; (b) ice velocity $u(x, t = 0)$. Blue lines show steady-state profiles. Inset in panel (a) shows a close-up of the vicinity of the grounding lines.

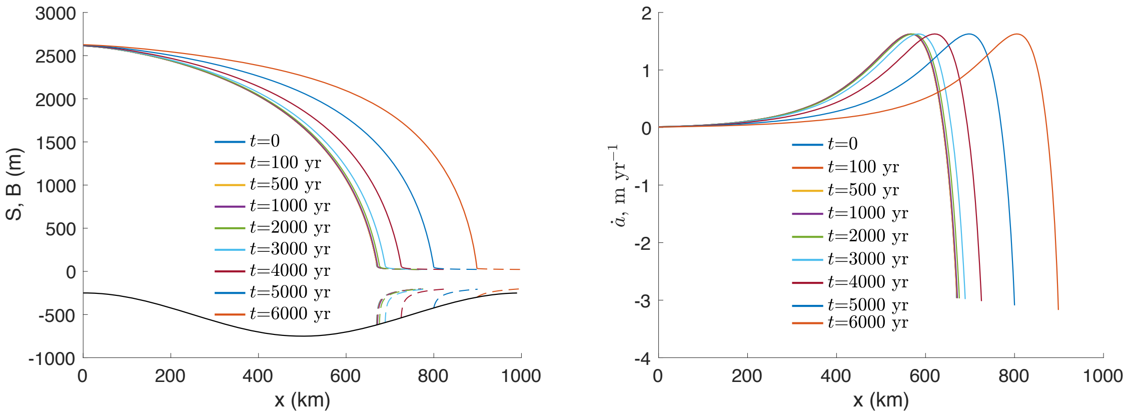

(a) S, B (m)

(b) $\dot{a}$ (m yr^{-1})

Figure 6: Evolution of the unstable ice sheet. (a) the ice-sheet configuration; (b) the corresponding accumulation/ablation rate $\dot{a}(T_S(S))$. Evolution of the grounding line is shown in Fig. 3a of the main text. Note different time intervals for the first three snapshots.

References

1. COMSOL. *Reference manual*. COMSOL, Boston, MA, (2022).

2. Powell, M. The BOBYQA algorithm for bound constraint optimization without derivatives. Technical Report NA06, DAMTP, (2009).

3. Sergienko, O. V. and Wingham, D. J. Bed topography and marine ice sheet stability. *Journal of Glaciology* **68**(267), 124–138 (2022), doi:10.1017/jog.2021.79.

4. Linden, H. Leighton's bounds for Sturm-Liouville eigenvalues with eigenvalue parameter in the boundary conditions. *Journal of Mathematical Analysis and Applications* **156**(2), 444 – 456 (1991), doi:10.1016/0022-247X(91)90408-R.

5. Kittel, C., Amory, C., Agosta, C., Jourdain, N. C., Hofer, S., Delhasse, A., Doutreloup, S., Huot, P.-V., Lang, C., Fichefet, T., and Fettweis, X. Diverging future surface mass balance between the Antarctic ice shelves and grounded ice sheet. *The Cryosphere* **15**(3), 1215–1236 (2021),doi:10.5194/tc-15-1215-2021.

6. Fettweis, X., Box, J. E., Agosta, C., Amory, C., Kittel, C., Lang, C., van As, D., Machguth, H., and Gallée, H. Reconstructions of the 1900–2015 Greenland ice sheet surface mass balance using the regional climate MAR model. *The Cryosphere* **11**(2), 1015–1033 (2017), doi:10.5194/tc-11-1015-2017.
